# Effects of Secondary Metabolites of Rice on Brown Planthopper and Its Symbionts

**DOI:** 10.3390/ijms25010386

**Published:** 2023-12-27

**Authors:** Ziyuan Deng, Chengling Lai, Jun Zhang, Fan Sun, Danting Li, Peiying Hao, Xuping Shentu, Kun Pang, Xiaoping Yu

**Affiliations:** Zhejiang Provincial Key Laboratory of Biometrology and Inspection & Quarantine, College of Modern Science and Technology, China Jiliang University, Hangzhou 310018, China; dzy13777583859@163.com (Z.D.); lchenglai@163.com (C.L.); zhangjun199909@163.com (J.Z.); sunfan20238@163.com (F.S.); lidanting@cjlu.edu.cn (D.L.); haopeiy@163.com (P.H.); stxp@cjlu.edu.cn (X.S.)

**Keywords:** brown planthopper, secondary metabolites, symbionts, methyl jasmonate

## Abstract

The brown planthopper *Nilaparvata lugens* (Stål) (BPH) is a main rice pest in China and many other Asian countries. In the control of BPH, the application of insect-resistant rice has proven to be quite effective. Secondary metabolites are essential weapons in plants’ defense against phytophagous insects. Studies have found that differences in the content of secondary metabolites play a crucial role in determining whether rice exhibits resistance or susceptibility to BPH. Simultaneously, symbionts are essential to the BPH. Nevertheless, there is limited research on the impact of secondary metabolites on the symbionts within BPH. Therefore, investigating the influence of secondary metabolites on both BPH and their symbionts is significant for the control of BPH. In this experiment, newly emerged female adults of BPH were fed artificial diets containing 10 different secondary metabolites. The results indicated that methyl jasmonate had inhibitory effects on the survival rate, weight gain, and reproductive capacity of BPH. Using qPCR methods, it was discovered that the number of symbiotic fungi (*Ascomycetes* symbionts) within BPH significantly decreased under methyl jasmonate stress. In conclusion, this experiment has preliminarily revealed the inhibitory effects of methyl jasmonate on BPH and its symbionts, demonstrating its potential for controlling BPH.

## 1. Introduction

The brown planthopper (BPH) is an injurious pest that sucks the juice of rice [1,2]. When the BPH experiences a significant outbreak, it can cause incalculable damage to rice production [3]. Research has shown that secondary metabolites in plant tissues are important in plant defense mechanisms against the BPH [4,5]. Simultaneously, throughout the evolutionary history of the BPH, symbionts play a crucial role in its normal life activities. However, there is limited research on the impact of secondary metabolites on the symbionts within the BPH. Therefore, investigating the influence of secondary metabolites, especially on the symbionts, holds significant importance.

The concept of plant secondary metabolites was first introduced by Kossel [6] in 1891. Among plant’s defense mechanisms, the synthesis of secondary metabolites within plants plays a significant role [7,8]. For example, quercetin, as a type of flavonoid compound, is primarily extracted from the rootstock of the ginger family plant [9]. It has toxic effects on insects [10]. For instance, quercetin exhibits a significant toxic effect on the sugar beet moth, and it also has insecticidal properties against the gypsy moth, *Lymantria dispar* [11,12]. Kaempferol is a natural flavonoid compound initially separated from the leaves and stems of the Moringa oleifera plant. Kaempferol exists mainly in plants in the form of glycosides [13]. Its content is not evenly distributed. Studies have found kaempferol in the fruit peel of the persimmon [14], but it is absent in the fruit pulp. Kaempferol has the highest content in the fruit peel of peanuts, and this content also varies among different peanut varieties [15,16]. Kaempferol exhibits antifungal activity [17,18], including inhibiting the growth of black mold and the synthesis of aflatoxins [19]. Apigenin, a trihydroxy flavonoid, is commonly found in various vegetables and has no toxic effects on humans and animals. It possesses antibacterial properties. Phenol oxidase in insects is vital for them [20,21]. Apigenin can inhibit the activity of phenol oxidase, thereby suppressing the sugar beet moth larvae [22]. Quercetin, a flavonoid compound, has significant inhibitory effects on the growth of cotton bollworms [23], tobacco budworms [24], and southern armyworms [25], affecting their survival. Salicylic acid is a simple phenolic compound that can induce resistance in plants against various fungi [26]. Benzyl benzoate has a notable toxic effect on the larvae and adults of the long-legged blood-sucking tick [27]. Anthocyanin, glycoside derivatives, are present in high levels in cotton leaves during the bud stage, and higher anthocyanin levels correlate with increased resistance of cotton to the green mirid bug [28]. Gallic acid is a natural organic phenolic acid that exhibits both polyphenolic and acidic properties [29,30,31]. It serves as an important secondary metabolite in plant resistance against phytophagous insects [32,33]. Gallic acid can inhibit the growth and development of wheat aphids [34] and reduce the activity of detoxifying enzymes in the Poplar longhorn beetle [35], affecting its survival. Methyl jasmonate can induce plants to produce toxic compounds [36,37] against insects, conferring insect resistance to the plant [38]. Naringenin, as a flavonoid compound, inhibits the activity of phenol oxidase in the diamondback moth, thereby affecting the insect [39].

In the continuous evolutionary process of insects, a mutually beneficial symbiotic relationship has formed between their internal symbionts, as observed in aphids, leafhoppers, and others [40,41,42]. The BPH also harbors a substantial number of symbionts, primarily distributed in the abdominal fat body and intestinal tissues [43,44,45]. Symbionts can be classified into two main categories: symbiotic fungi, primarily consisting of yeast-like symbionts (YLS), dominated by *Arsenophonus* in the male-killing fungus genus and *Wolbachia* [41,46]. In the 1960s, Nasu isolated YLS from the fat body of BPH [47]. With the advancement of scientific technology, an increasing number of symbiotic fungi have been discovered and identified in various tissues of the BPH, such as the fat body, intestines, and ovaries [48,49]. Noda et al. [50] first separated and proved the Noda fungus by applying the 18S rDNA sequence. Symbionts are essential to the BPH [51,52]. YLS is involved in the synthesis of sterols in BPH and influences the synthesis of essential amino acids [53,54]. *Arsenophonus* in the male-killing fungus genus is vital to the synthesis of B-group vitamins in the BPH [55]. *Wolbachia* promotes BPH egg-laying by producing vitamins B2 and B7 [52]. Simultaneously, symbionts contribute to the development of resistance in the BPH. *Wolbachia* helps the BPH adapt to Mudgo-resistant rice [56]. *Arsenophonus* assists the BPH in resisting the invasion of the pathogenic fungus Metarhizium flavoviride and plays a crucial role in the resistance to the chemical pesticides of BPH [57,58]. Thus, symbionts within the BPH participate in many life activities, playing a significant role in its overall biology. Therefore, influencing the symbionts within the BPH provides a feasible approach to controlling and preventing infestations of BPH.

In conclusion, plants can utilize secondary metabolites to resist the invasion of BPH. However, whether secondary metabolites, in the specific defense mechanism, achieve their defensive purpose by suppressing beneficial symbionts within the BPH has not been clearly elucidated. Recent studies have indicated that sakuranetin can defend rice against the BPH by inhibiting the growth of the YLS within the BPH [59]. Consequently, it is vital to study whether other secondary metabolites have inhibitory effects on the symbionts. In this experiment, ten secondary metabolites were selected to explore their effects on the BPH and its symbionts, especially *Ascomycetes* symbionts. In this study, the effects of 10 secondary metabolites, including kaempferol, luteolin, apigenin, quercetin, salicylic acid, benzyl benzoate, anthocyanin, gallic acid, methyl jasmonate, and naringenin, on the BPH and its symbionts were studied.

## 2. Results

### 2.1. Results of the Effect of Secondary Metabolites on the Brown Planthopper

#### 2.1.1. Mortality Rate 

**Figure 1:**
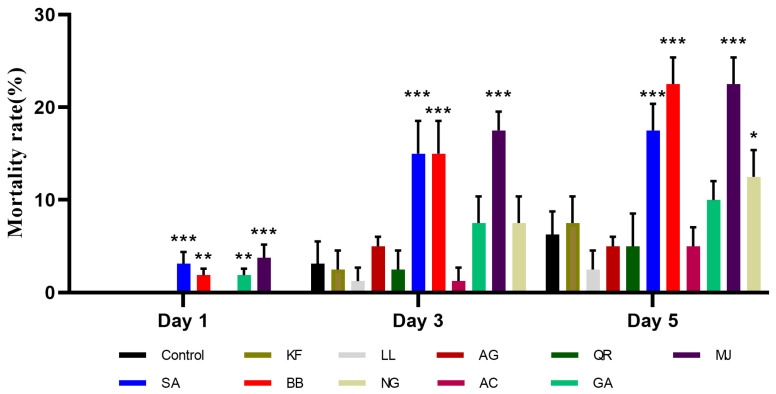
Mortality rate of newly emerged female adults of BPH at 1, 3, and 5 days under different secondary metabolites stress (%). KF: kaempferol; LL: luteolin; AG: apigenin; QR: quercetin; MJ: methyl jasmonate; SA: salicylic acid; BB: benzyl benzoate; NG: naringenin; AC: anthocyanin; GA: gallic acid. *, *p* < 0.05; **, *p* < 0.01; ***, *p* < 0.001, comparison with control group (Dunnett test).

According to Figure 1, when treated for 1 day, there were significant differences in mortality rates between the four treatment groups: methyl jasmonate, salicylic acid, benzyl benzoate, and gallic acid, compared to the control group. Among these, the group treated with methyl jasmonate exhibited the highest mortality rate at 3.75%, followed by the salicylic acid group with a mortality rate of 3.13%. The mortality rates for the benzyl benzoate and gallic acid treatment groups were both 1.88%. The mortality rates for the other treatment groups were all 0.00%. After 3 days of treatment, the mortality rates for the three treatment groups—methyl jasmonate, salicylic acid, and benzyl benzoate—still showed clearer distinction. Among these, the group treated with methyl jasmonate had the highest mortality rate at 17.50%. The mortality rates for the other secondary metabolite treatment groups and the control group also displayed an increasing trend. By day 5, the mortality rates for the four treatment groups—methyl jasmonate, benzyl benzoate, salicylic acid, and naringenin—were significant. Among these, both the methyl jasmonate and benzyl benzoate treatment groups had the highest mortality rate at 22.50%, while the salicylic acid treatment group had a mortality rate of 17.50%. The mortality rates for the other treatment groups also increased to some extent.

From the experimental results, it is evident that the mortality rates in the methyl jasmonate, benzyl benzoate, and salicylic acid treatment groups consistently exhibited significant differences, with the methyl jasmonate treatment group having the highest mortality rate and the most significant impact on BPH survival. 

#### 2.1.2. Weight Gain Rate

**Figure 2:**
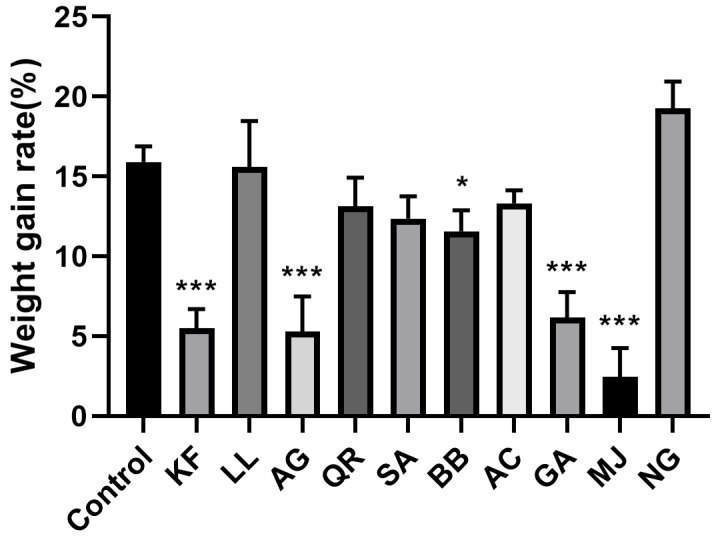
Weight gain rate of newly emerged female adults of BPH after 48 h under different secondary metabolites stress (%). KF: kaempferol; LL: luteolin; AG: apigenin; QR: quercetin; MJ: methyl jasmonate; SA: salicylic acid; BB: benzyl benzoate; NG: naringenin; AC: anthocyanin; GA: gallic acid. *, *p* < 0.05; ***, *p* < 0.001, comparison with control group (Dunnett test).

As shown in Figure 2, BPH fed with an artificial diet containing naringenin for 48 h had the highest weight gain rate. It shows a potential promotion of weight gain in BPH. BPH fed with an artificial diet containing methyl jasmonate, apigenin, kaempferol, gallic acid, and benzyl benzoate exhibited a delayed weight gain rate when compared to the control group. Among these secondary metabolites, methyl jasmonate, apigenin, kaempferol, gallic acid, and benzyl benzoate had inhibition effects on the growth of newly emerged female adults of BPH. Among these five secondary metabolites, BPH subjected to methyl jasmonate stress had the slowest weight gain rate, increasing by only 2.44% after 48 h.

#### 2.1.3. Fecundity

**Figure 3:**
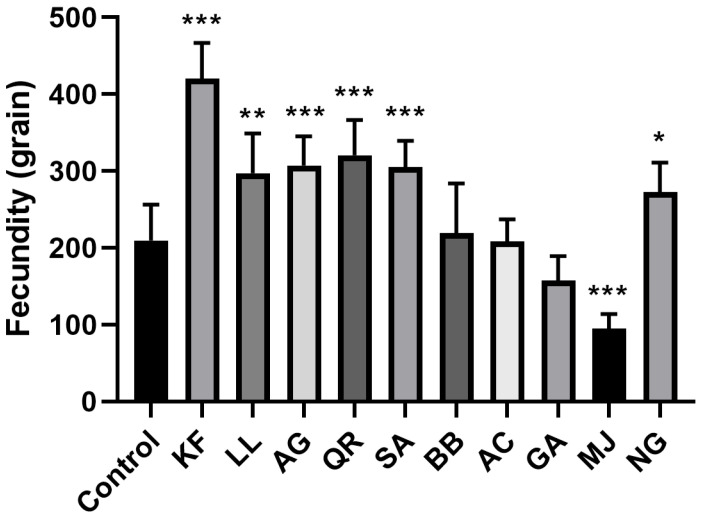
The fecundity of female adults of BPH under stress of different secondary metabolites (grains). KF: kaempferol; LL: luteolin; AG: apigenin; QR: quercetin; MJ: methyl jasmonate; SA: salicylic acid; BB: benzyl benzoate; NG: naringenin; AC: anthocyanin; GA: gallic acid. *, *p* < 0.05; **, *p* < 0.01; ***, *p* < 0.001, comparison with control group (Dunnett test).

As depicted in Figure 3, BPH fed with an artificial diet containing kaempferol had the highest total egg production, reaching 421 eggs, significantly higher than the control group with 210 eggs. BPH fed with an artificial diet containing methyl jasmonate had the lowest total egg production, with only 95 eggs, displaying a significant inhibitory effect.

#### 2.1.4. The Number of Symbionts

Based on earlier experiments assessing the mortality rate, weight gain rate, and fecundity of BPH under the stress of different secondary metabolites, five secondary metabolites with notably significant effects were selected: methyl jasmonate, benzyl benzoate, apigenin, quercetin, and gallic acid. Newly emerged female adults of BPH were fed with artificial diet solutions containing these five secondary metabolites, respectively.

**Figure 4:**
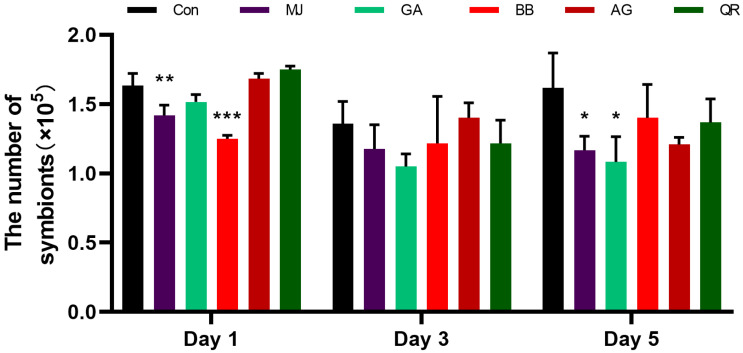
The number of symbionts in female adults of BPH at 1, 3, and 5 days under different secondary metabolites stress (%). KF: kaempferol; LL: luteolin; AG: apigenin; QR: quercetin; MJ: methyl jasmonate; SA: salicylic acid; BB: benzyl benzoate; NG: naringenin; AC: anthocyanin; GA: gallic acid. *, *p* < 0.05; **, *p* < 0.01; ***, *p* < 0.001, comparison with control group (Dunnett test).

According to Figure 4, after 1 day of feeding, the group treated with benzyl benzoate had the fewest symbionts within female adults of BPH compared to the control group, showing highly significant differences. The group treated with methyl jasmonate had the next lowest count, displaying significant differences. After 3 days of feeding, the group treated with gallic acid had the fewest symbionts within female adults of BPH compared to the control group. The group treated with methyl jasmonate had the next lowest count. After 5 days of feeding, both the gallic acid and the methyl jasmonate showed obvious inhibition effects on symbionts.

### 2.2. The Symbionts of Ascomycetes Symbionts Were Quantitatively Detected by Real-Time Quantitative Polymerase Chain Reaction

qPCR detection method was used for quantifying *Ascomycetes* symbionts within the intestine of BPH. Quantitative PCR (qPCR) was employed to measure the copy numbers by utilizing a standard curve. Experimental results showed that after 1 day of feeding, the intestine of BPH under the stress of methyl jasmonate had more *Ascomycetes* symbionts compared to the control group. However, after 3 and 5 days, the methyl jasmonate group had a lower quantity of *Ascomycetes* symbionts within the intestine, and the number of symbionts in the methyl jasmonate-treated group continued to decrease. 

**Figure 5:**
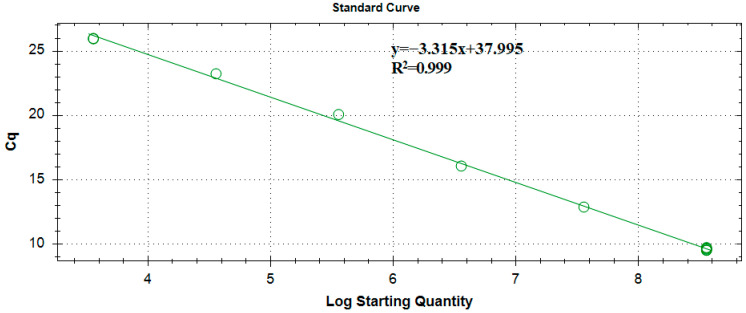
The standard curve of *Ascomycetes* symbionts Real-time Quantitative Polymerase Chain Reaction products.

As shown in Figure 5, it is the standard curve of Ascomycetes symbionts Real-time Quantitative Polymerase Chain Reaction products.

**Figure 6:**
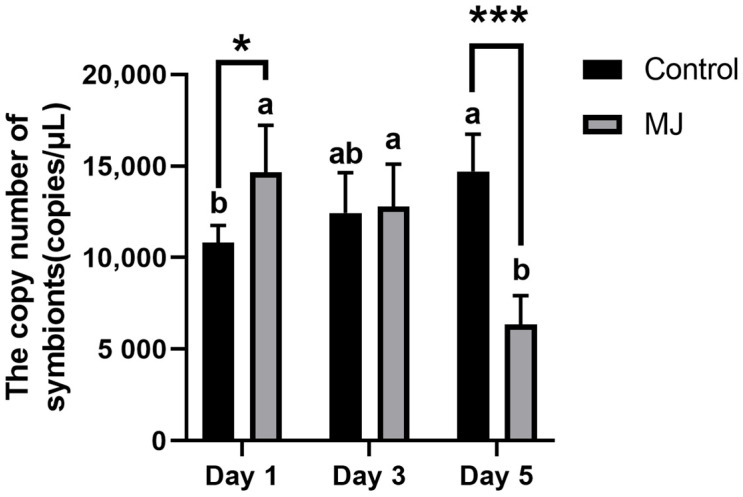
Quantitative detection of copy number of *Ascomycetes* symbionts in the intestine of BPH by qPCR (copies/μL). *, *p* < 0.05; ***, *p* < 0.001, MJ group and control group were compared on the same day (*t* test). There are significant differences between different lowercase letters (comparison between different days of the same treatment (control or MJ), *p* < 0.05, one-way ANOVA, and multiple comparisons of Tukey).

As shown in Figure 6 qPCR detection method was used for quantifying Ascomycetes symbionts within the intestine of BPH. Quantitative PCR (qPCR) was employed to measure the copy numbers by utilizing a standard curve. Experimental results showed that after 1 day of feeding, the intestine of BPH under the stress of methyl jasmonate had more quantity of Ascomycetes symbionts compared to the control group. However, after 3 and 5 days, the methyl jasmonate’s group had a lower quantity of Ascomycetes symbionts within the intestine, and the quantity of symbionts in the methyl jasmonate-treated group continued to decrease. 

**Figure 7:**
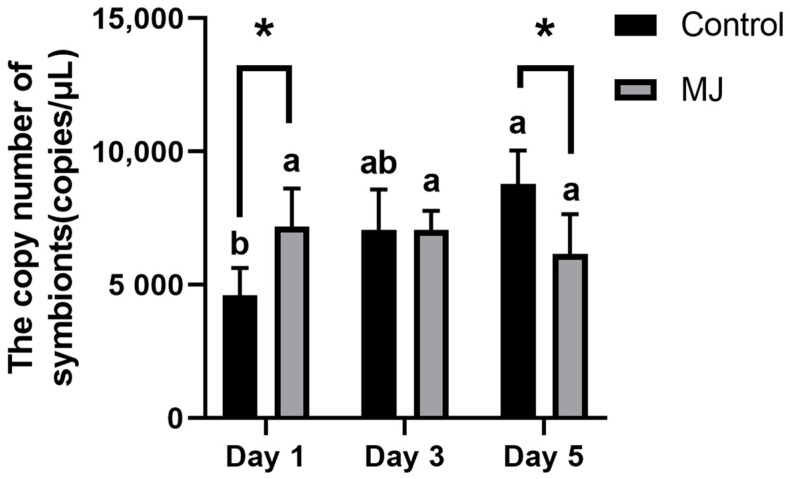
Quantitative detection of copy number of *Ascomycetes* symbionts in the hemolymph of BPH by qPCR (copies/μL). *, *p* < 0.05, MJ group and control group were compared on the same day (*t* test). There are significant differences between different lowercase letters (comparison between different days of the same treatment (Control or MJ), *p* < 0.05, one-way ANOVA, and multiple comparisons of Tukey).

As shown in Figure 7 quantitative PCR (qPCR) was conducted to measure *Ascomycetes* symbionts within the hemolymph of BPH, and copy numbers were calculated using a standard curve. Experimental results revealed that after 1 day of feeding, BPH under the stress of methyl jasmonate had more quantity of *Ascomycetes* symbionts in their hemolymph compared to the control group. However, after 3 and 5 days, the group treated with methyl jasmonate had a lower quantity of *Ascomycetes* symbionts in their hemolymph, and the number of symbionts in the methyl jasmonate-treated group continued to decrease.

**Figure 8:**
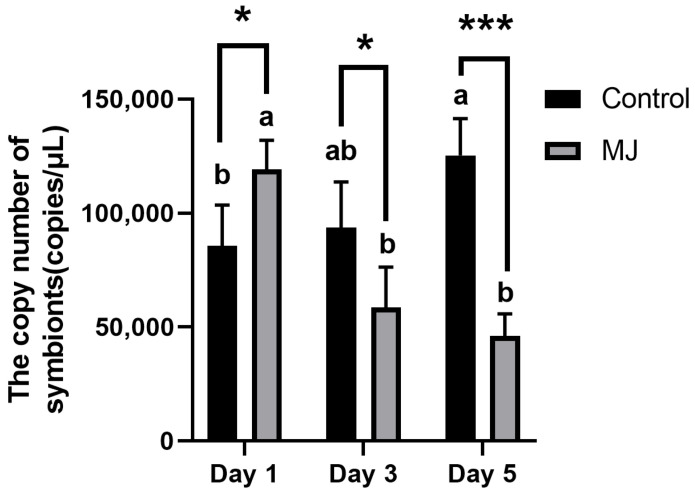
Quantitative detection of copy number of *Ascomycetes* symbionts in the fat body of BPH by qPCR (copies/μL). *, *p* < 0.05; ***, *p* < 0.001, MJ group and control group were compared on the same day (*t* test). There are significant differences between different lowercase letters (comparison between different days of the same treatment (Control or MJ), *p* < 0.05, one-way ANOVA, and multiple comparisons of Tukey).

As shown in Figure 8 quantitative PCR (qPCR) was employed to measure *Ascomycetes* symbionts within the fat body of BPH, and copy numbers were calculated using a standard curve. Experimental results demonstrated that after 1 day of feeding, BPH under the stress of methyl jasmonate had more quantity of *Ascomycetes* symbionts in their fat body compared to the control group. However, after 3 and 5 days, the group treated with methyl jasmonate had a lower quantity of *Ascomycetes* symbionts in their fat body, and the number of symbionts in the methyl jasmonate-treated group continued to decrease.

In summary, the analysis suggests that methyl jasmonate’s stress effect on BPH may manifest in its impact on the population of symbionts within the insects, thereby posing a threat to the survival of BPH.

## 3. Discussion

Secondary metabolites can also have lethal effects on insects, primarily through stomach and contact poisoning mechanisms [60]. Amygdalin induces insect mortality through stomach poisoning but lacks contact-killing properties [61]. Stomach poisoning can have different underlying principles of lethality. For instance, amygdalin primarily disrupts the insect’s intestine membrane system to induce poisoning [62], while berberine acts by damaging insect mid-intestine cells, thereby disrupting their neural transmission, leading to lethality [63,64]. On the other hand, eugenol causes the mortality of adult common pistachio psyllids (*Agonoscena pistaciae*) through contact killing [65]. Furthermore, some secondary metabolites can exhibit both stomach poisoning and contact-killing effects on insects. For example, feverfew extract has both stomach poisoning and contact-killing effects on the coconut husk aphid (*Pentalonia nigronervosa*) but only stomach poisoning effects on the coconut hispine beetle (*Brontispa longissima*). This phenomenon suggests that the same plant may exert different lethal effects on different insects [66,67]. In this experiment, the results of feeding indicate that methyl jasmonate, benzyl benzoate, and salicylic acid, these three secondary metabolites, have a significant impact on BPH mortality, with methyl jasmonate being the most potent in inducing lethality.

Secondary metabolites can inhibit insect health, prolonging the insect’s developmental period and decreasing its body weight [68,69,70,71]. Flavonoids, for instance, have a significant impact on insect growth and development. Quercetin affects the larval stage and pupation rate of the cabbage looper (*Trichoplusia ni*), influencing its normal growth and development [71]. Tannic acid reduces the activity of digestive enzymes within insects, weakening their digestive capabilities and preventing normal growth and development [72,73]. When caterpillars of the fall webworm (*Hyphantria cunea*) are treated with tannic acid, the molting process is entirely disrupted, and they cannot complete this process, leading to death by the fourth instar [74]. In this experiment, these four secondary metabolites—methyl jasmonate, apigenin, kaempferol, and gallic acid—have a significant inhibitory effect on the weight gain of BPH, with the slowest weight gain observed in the group subjected to methyl jasmonate stress. Additionally, the study explored the effect of ten kinds of secondary metabolites on the fecundity of BPH. The results show that BPH subjected to methyl jasmonate had the lowest fecundity, indicating an inhibitory effect. The results also show that BPH subjected to kaempferol had the highest fecundity, indicating a promoting effect. Recent studies have indicated that SeNPs exhibit a hormetic response (fitness increase at low dose) to male seed beetles [75]. We could explore whether kaempferol might have a similar effect on BPH. This aspect will be a focus and objective in our future research.

Secondary metabolites can impact the symbionts within insects. Regarding their antibacterial properties, secondary metabolites extracted from disease-resistant rice varieties, like quercetin, kaempferol, and anthocyanins, significantly inhibit the growth and spore germination of rice blast fungus (*Pyricularia grisea*) and rice bacterial leaf blight pathogen [76]. Research has shown that aconitine and nicotine can influence the dominant microbial community in the intestine of Dendrolimus superans larvae [77]. In this experiment, gallic acid and methyl jasmonate significantly reduced the population of symbionts within BPH. Building upon this, quantitative PCR was used to assess *Ascomycetes* symbionts in the intestine, hemolymph, and fat body of BPH subjected to methyl jasmonate stress. Comparing these groups with the control, it was found that methyl jasmonate has an inhibitory effect on *Ascomycetes* symbionts within BPH. However, the dynamic response of endosymbiont to methyl jasmonate has not been thoroughly investigated. This aspect will be a focus and objective in our future research.

In summary, methyl jasmonate exerts the strongest stress on BPH. It inhibits their survival, weight gain rate, fecundity, and the population of symbionts significantly compared to the control group. Methyl jasmonate also has an inhibitory effect on *Ascomycetes* symbionts.

## 4. Materials and Methods

### 4.1. Principal Reagent, Instruments, and Materials

#### 4.1.1. Experimental Reagent

Kaempferol, KF (Aladdin), luteolin, LL (Aladdin), apigenin, AG (Aladdin), quercetin, QR (Aladdin), salicylic acid, SA (Aladdin), benzyl benzoate, BB (Macklin), anthocyanin, AC (Macklin), gallic acid, GA (Aladdin), methyl jasmonate, MJ (Macklin), naringenin, NG (Aladdin), KOH (Aladdin), 10 × PBS, deionized water (Sterile) (Biosharp), yeast extract for laboratory use only (OXOID), D-anhydrous glucose (Solarbio), agar powder (Solarbio), Peptone (Solarbio), LB broth (Hopebio), LB agar (Hopebio), anhydrous ethanol, TIANNamp Genomic DNA Kit (TIANGEN), SYBR Green Premix Pro Taq HS qPCR Kit (Accurate Biotechnology (Hunan) Co., Ltd., Hunan, China), SanPrep Column Plasmid Mini-Preps Kit (Sangon Biotech, Shanghai, China)

#### 4.1.2. Reagent for Artificial Feeding Solution

Glycine, L-alanine, L-arginine hydrochloride, L-asparagine, L-aspartic acid (Sigma), L-cysteine, L-cystine hydrochloride, γ-amino butyric acid, L-glutamic acid, L-glutamine, L-histidine, L-methionine, L-isoleucine, L-leucine, L-proline, L-lysine hydrochloride, L-phenylalanine, L-serine, L-threonine, L-tryptophane, L-tyrosine, biotin, calcium pantothenate, choline chloride, folic acid, inositol, nicotinic acid, riboflavin, thiamine hydrochloride, ascorbic acid (Sigma, Kanagawa, Japan), pyridoxine hydrochloride (Aladdin), CaCl_2_·2H_2_O (Macklin), CuCl_2_·2H_2_O (Macklin), FeCl_3_·6H_2_O (Aladdin), L-valine (Solarbio), MnCl_2_·4H_2_O (Macklin), ZnCl_2_ (Aladdin), MgCl_2_·6H_2_O (Aladdin), KH_2_PO_4_ (Aladdin), sucrose (Aladdin).

#### 4.1.3. Instruments and Materials

Electronic balance (Sartorius), vortex oscillators, ultra-clean table (Boxum), ultra-pure water system, glass double tube, parafilm sealing film, shade cloth, Millipore disposable filter (0.45 μm), glass test tube, gauze, stereomicroscope (Nikon), dissecting forceps, grinding bead, grinding rod, optical microscope (Nikon), blood cell counting plate, HYG-full temperature shaker cabinet, THZ-D table thermostatic oscillator, autoclave, 250 mL conical flask, sterile drug sensitive tablets.

### 4.2. Test Insect

Adults of BPH were collected from rice fields in Hangzhou, China (30° N, 120° E). The BPH used in the experiment belonged to a laboratory-established population, which was reared in insect cages and artificial climate chambers produced by Jiangnan Instrument Manufacturing Company. The rearing temperature for BPH was maintained at 24 °C~26 °C, with a relative humidity of 55~65%, and a light regime of 16 h light and 8 h dark (L:D = 16:8). The rice variety used to feed the BPH was Xiushui 134, with seedlings exceeding 3 cm in height, and they had been reared on Xiushui 134 rice plants for over 25 generations. Rice seeds for feeding BPH were evenly spread in acrylic plates with damp perlite after two days of soaking, and during the cultivation process, only plain water was used without the addition of any other substances. The acrylic plates containing the rice seeds were placed in meshed insect-rearing cages and watered daily until the seedlings reached a height of over 3 cm and were suitable for feeding the BPH. BPH was propagated by introducing 100 female and 50 male adults into new seedlings for mating.

### 4.3. Preparation of Artificial Feeding Liquid

Following the preparation method of artificial diet D-97 as described by Fu Qiang [78], with specific components outlined in Section 4.1.2. The preparation volume for the artificial diet is 100 mL. First, weigh all the amino acids to make a 2× stock solution. Weigh all vitamins, excluding ascorbic acid, to make a 10× stock solution. Weigh all inorganic salts, excluding FeCl_3_·6H_2_O, to make a 100× stock solution. After preparation, if not used immediately, store it in a −20 °C freezer for later use. Take a beaker and sequentially add the components, such as the amino acid stock solution, sucrose, MgCl_2_·6H_2_O, KH_2_PO_4_, and FeCl_3_·6H_2_O, in the given order while stirring until completely dissolved. Then, continue adding the vitamin solution, inorganic salt solution, and 10× ascorbic acid solution in that order, stirring until fully dissolved. Adjust the pH of the prepared artificial diet to 6.8 using a 4% KOH solution, and then dilute the mixture to 100 mL with ultrapure water. Finally, in a laminar flow cabinet, filter the artificial diet through a Millipore disposable filter (0.45 μm) to sterilize it, divide it into portions, and store it in a −20 °C freezer for later use.

### 4.4. Preparation of Artificial Feeding Solution Containing Secondary Metabolites

The artificial diet containing secondary metabolites was prepared by dissolving the 10 secondary metabolites used in the experiment (kaempferol, apigenin, luteolin, quercetin, salicylic acid, benzyl benzoate, anthocyanin, gallic acid, methyl jasmonate, and naringenin) separately in ultrapure water. The final stock solution concentrations for each secondary metabolite were adjusted to 200 mg/mL based on their maximum solubility in water and the maximum solubility in the artificial diet. The same dose of DNase/RNase-free ddH_2_O with other secondary metabolites was added into the artificial feeding solution as the experimental material of the control group. If not used immediately, the prepared solutions can be stored at −20 °C in the refrigerator.

### 4.5. The Brown Planthopper Was Fed with Artificial Feeding Solution

The method for feeding BPH with an artificial diet was adapted from Fu et al. [78], with some improvements as follows: double-ended glass tubes (15 cm in length, 2.5 cm in diameter) were used as the living places for BPH. Before feeding, the glass tubes were thoroughly cleaned and dried. One end of each double-ended tube was sealed with Parafilm. Twenty newly emerged female adults of BPH were aspirated using a pipette and blown into the double-ended tube, ensuring that none escaped, and then the other end of the tube was sealed. Subsequently, 20 μL of the artificial diet was pipetted onto the Parafilm at one end of the tube. The Parafilm was then doubly sealed with stretched layers of Parafilm, following the same procedure for the other end. The sealed double-ended tubes were placed flat and covered with a dark-colored fabric (black or brown cotton cloth), ensuring it was in the dark. The BPH is fed on the diet by piercing through the Parafilm membrane. The fresh diet was replaced every 24 h.

### 4.6. Bioassay of the Brown Planthopper under Secondary Metabolite Stress

#### 4.6.1. Mortality

The subjects of this experiment were newly emerged female adults of BPH. Each secondary metabolite constituted an experimental group, with four replicates in each group. Each group was fed 20 newly emerged female adults of BPH. The number of dead BPH within each group was counted and removed every 24 h, and a fresh artificial diet corresponding to the respective secondary metabolite was replaced until the end of the fifth day.

#### 4.6.2. Weight Gain Rate

The subjects of this experiment were newly emerged female adults of BPH. Each secondary metabolite constituted an experimental group, with four replicates in each group. Twenty newly emerged female adults of BPH were aspirated into 50 mL centrifuge tubes in each group, and their initial average weight was measured. Then, they were transferred to the feeding chambers and fed with artificial diets containing different secondary metabolites for 48 h. After the 48 h feeding period, the mortality of the BPH was observed, the surviving individuals were aspirated and weighed again, and the average weight for each group was calculated.

#### 4.6.3. Fecundity

The subjects of this experiment were newly emerged female adults of BPH. Based on the mortality rate data for each secondary metabolite, 60–80 newly emerged female adults of BPH were divided into 3–4 groups and placed in the feeding chambers, where they were fed with artificial diets containing different secondary metabolites for 48 h. For each secondary metabolite, 30 glass test tubes were prepared, and fresh 5–7 cm rice seedlings were placed in the tubes with plain water to ensure the normal growth of rice seedlings. The water in the tubes contained no additional substances. Subsequently, the surviving newly emerged female adults of BPH after the 48 h feeding period were introduced into the glass tubes with the prepared rice seedlings. Each tube contained one female, and a male adult of BPH that had emerged 48 h earlier was introduced to facilitate mating. Finally, the openings of the tubes were sealed with gauze. Every 24 h, the BPH in the glass tubes was aspirated out, the rice seedlings were replaced with fresh ones, and the BPH was reintroduced. This process was repeated until the death of the female adults of BPH. The rice seedlings from the glass tubes were dissected to count the eggs, and the data were recorded until the death of the female adults of BPH.

#### 4.6.4. The Quantity of Symbionts

The subjects of this experiment were newly emerged female adults of BPH. Each group had 20 newly emerged female adults of BPH introduced into the feeding chambers and fed with artificial diets containing different secondary metabolites. Each secondary metabolite constituted an experimental group, and each group had three replicates. Counted the symbionts on the first, third, and fifth days. Detailed procedures involved taking five surviving BPH for each group, disinfecting their surface with alcohol, rinsing with PBS solution, adding 500 μL of PBS solution, and appropriate grinding beads. After grinding, the suspension was vortexed, and 10 μL of the liquid was drawn into a hemocytometer for symbiont counting. The calculation formula used was symbiont number/μL = average number of symbionts per small square × 400 × 10 × dilution factor.

#### 4.6.5. Data Statistics and Analysis

Data were statistically analyzed using SPSS 27 and graphed using GraphPad Prism 6.01 (GraphPad Software Inc., La Jolla, CA, USA). Data are presented as mean ± standard deviation. Differences in means were analyzed using the one-way ANOVA and multiple comparisons of the Dunnett test.

### 4.7. Effect of Methyl Jasmonate on Symbionts in Nilaparvata Lugens

#### 4.7.1. Sample Collection of the Brown Planthopper under Methyl Jasmonate Stress

Collection of BPH samples under methyl jasmonate stress artificial diets containing methyl jasmonate were prepared, and newly emerged female adults of BPH were fed with this artificial diet. The feeding groups were divided into three categories: 1-day feeding group, 3-day feeding group, and 5-day feeding group, with each group consisting of 60 BPH. These planthoppers were placed in three separate feeding chambers, and each treatment duration had three replicates.

#### 4.7.2. Acquisition of Intestinal Tract, Fat Body, and Hemolymph from the Brown Planthopper

For each treatment group, 30 surviving BPH were collected and exposed to 75% alcohol for 10 min to disinfect them. The alcohol was then removed using a pipette, and the insects were rinsed with 75% alcohol for three minutes, twice. After rinsing, they were washed with 1× PBS solution for three minutes, repeated three times, ensuring thorough removal of any residual alcohol. Once the rinsing was complete, a clean glass slide was taken, and 1× PBS solution was pipetted onto it, creating a grid of 20 drops (2 rows of 10 drops). Each BPH was placed in a drop of the 1× PBS solution on the glass slide. The glass slides, each containing a BPH specimen, were then examined under a microscope, and the intestine, fat body, and hemolymph were collected and placed in a pre-prepared 1× PBS solution.

#### 4.7.3. Extraction of Symbiotic DNA

After collection, DNA was extracted following the instructions provided in the TIANNamp Genomic DNA Kit. If DNA extraction was not performed immediately, it was stored in a −80 °C freezer, but not for an extended period.

#### 4.7.4. Quantification of the Symbionts by Real-Time Quantitative Polymerase Chain Reaction 

Using *Ascomycetes* symbiont-positive plasmids preserved by our research group as materials, plasmid extraction was carried out with the SanPrep Column Plasmid Mini-Preps Kit. After measuring the concentration of the extracted plasmids, the copy number was calculated using the formula: (6.02 × 10^23^) × (ng/μL × 10^−9^)/(DNA length × 660) = copies/μL. Subsequently, the plasmids were subjected to gradient dilution using DNase/RNase-free water, resulting in dilutions of 10^8^, 10^7^, 10^6^, 10^5^, 10^4^, and 10^3^ copies/μL, which served as templates for the standard curve experiment. The extracted DNA samples were used as experimental templates. The specific primer sequences for *Ascomycetes* symbionts are detailed in Table 1, and the reaction system is outlined in Table 2. Refer to Table 3 for the reaction conditions. The experiment was conducted, and reaction results were observed. Statistical analysis and graphical representation of data were performed using GraphPad Prism 6.01 (GraphPad Software Inc., La Jolla, CA, USA). A *t*-test was utilized to compare different treatments on the same day, while one-way ANOVA and Tukey’s multiple comparisons were applied to compare different days under the same treatment.

## 5. Conclusions

This study investigated the effects of ten secondary metabolites, including kaempferol, luteolin, apigenin, quercetin, salicylic acid, benzyl benzoate, anthocyanin, gallic acid, methyl jasmonate, and naringenin, on the survival, weight gain rate, and fecundity of the BPH. The results showed that methyl jasmonate inhibits BPH. Using qPCR methods, it was discovered that the number of symbiotic fungi (*Ascomycetes* symbionts) within BPH significantly decreased under methyl jasmonate stress. This lays the foundation for future research to clarify the stress mechanism of methyl jasmonate on symbionts in BPH and provides theoretical support for the future development of bacterial inhibition for pest control technology.

## Figures and Tables

**Table 1:** Specific primer sequences of *Ascomycetes* symbionts.

Symbiont	Primers	Sequences (5′→3′)	Amplified Product Length
*Ascomycetes symbionts*	AsF	GTCGTAGTCTTAACCATAA	145
	AsR	CTTCCGTCAATTTCTTTAAG	

**Table 2:** Reaction system.

Component Name	Volume (μL)
2 ×SYBR Green Pro Taq HS Premix	5.0
ddH_2_O	3.6
Upstream primer	0.2
Downstream primer	0.2
Template DNA	1.0
Total	10.0

**Table 3:** Reaction condition of qPCR.

Reaction Temperature (°C)	Reaction Time	Cycle Number
95	30 s	1
95	5 s	40
56	30 s	

## Data Availability

All data are included in figures or can be obtained by contacting the corresponding author.
